# Supplementary figures and images for: DiscoTope-3.0: improved B-cell epitope prediction using inverse folding latent representations
Source: Front Immunol. 2024 Feb 8;15:1322712. doi: 10.3389/fimmu.2024.1322712 (PMC10882062; doi:10.3389/fimmu.2024.1322712)

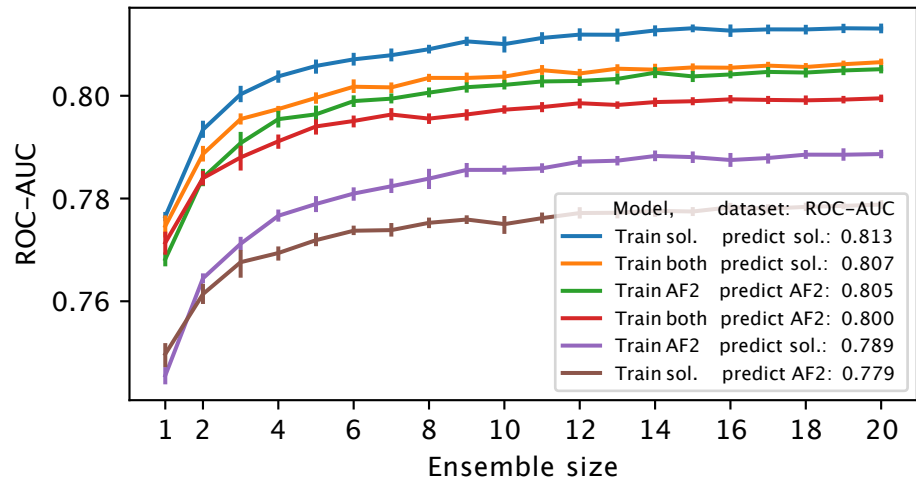

Supplement: Supplementary Figure 1 — Validation set performance up to ensemble size 20. Validation set gain in AUC-ROC from ensembling the full-feature model. Performance graphs are shown for training on either experimentally solved, AlphaFold predicted or both structures, and then evaluated on either the solved or predicted structure validation set. [file DataSheet_1.zip › figures/S1_ablation_graph.pdf]

Length distribution

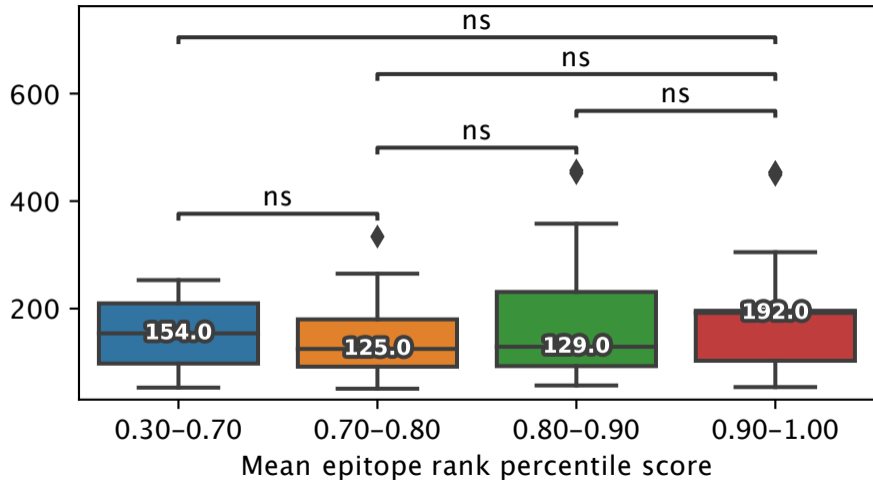

Supplement: Supplementary Figure 1 — Validation set performance up to ensemble size 20. Validation set gain in AUC-ROC from ensembling the full-feature model. Performance graphs are shown for training on either experimentally solved, AlphaFold predicted or both structures, and then evaluated on either the solved or predicted structure validation set. [file DataSheet_1.zip › figures/S4_length_plddt.pdf]

a) Solved to predicted

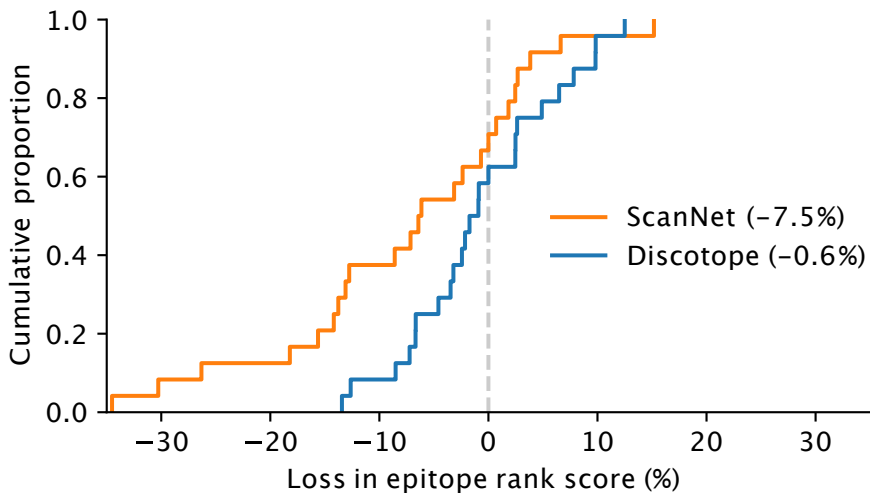

b) Solved to relaxed

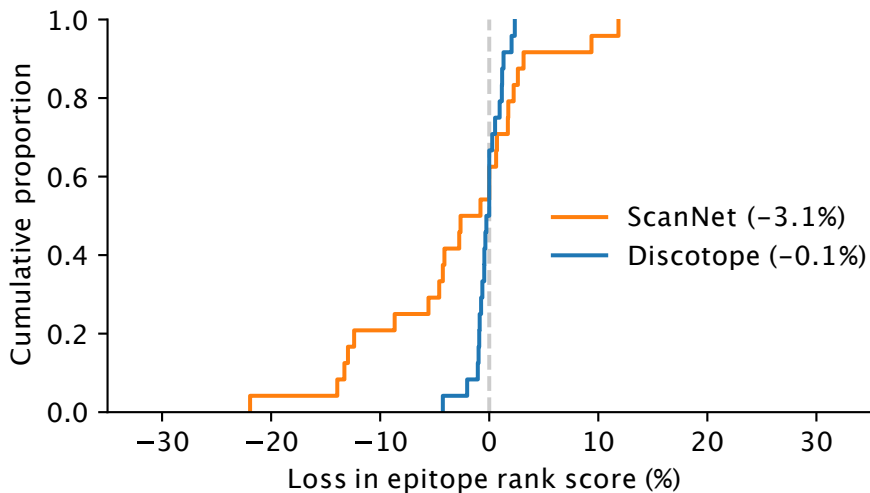

Supplement: Supplementary Figure 1 — Validation set performance up to ensemble size 20. Validation set gain in AUC-ROC from ensembling the full-feature model. Performance graphs are shown for training on either experimentally solved, AlphaFold predicted or both structures, and then evaluated on either the solved or predicted structure validation set. [file DataSheet_1.zip › figures/S5_pred_vs_solved.pdf]

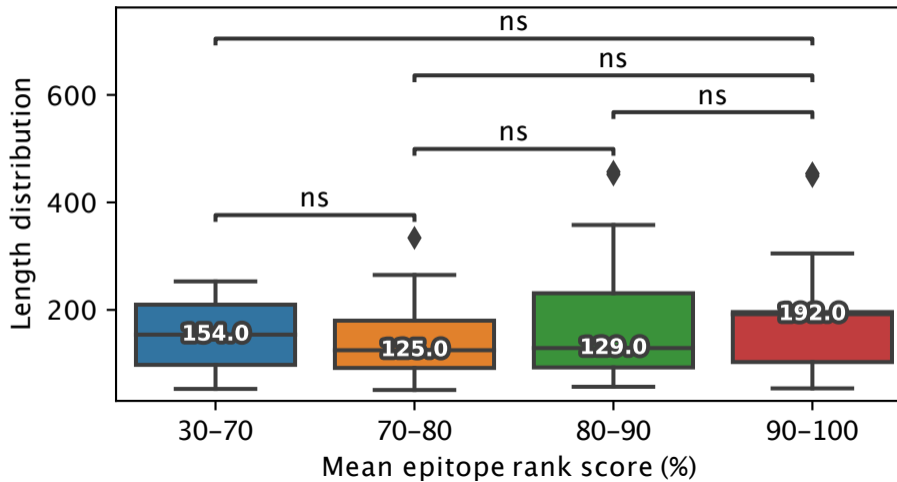

Supplement: Supplementary Figure 1 — Validation set performance up to ensemble size 20. Validation set gain in AUC-ROC from ensembling the full-feature model. Performance graphs are shown for training on either experimentally solved, AlphaFold predicted or both structures, and then evaluated on either the solved or predicted structure validation set. [file DataSheet_1.zip › figures/S5_length_plddt.pdf]

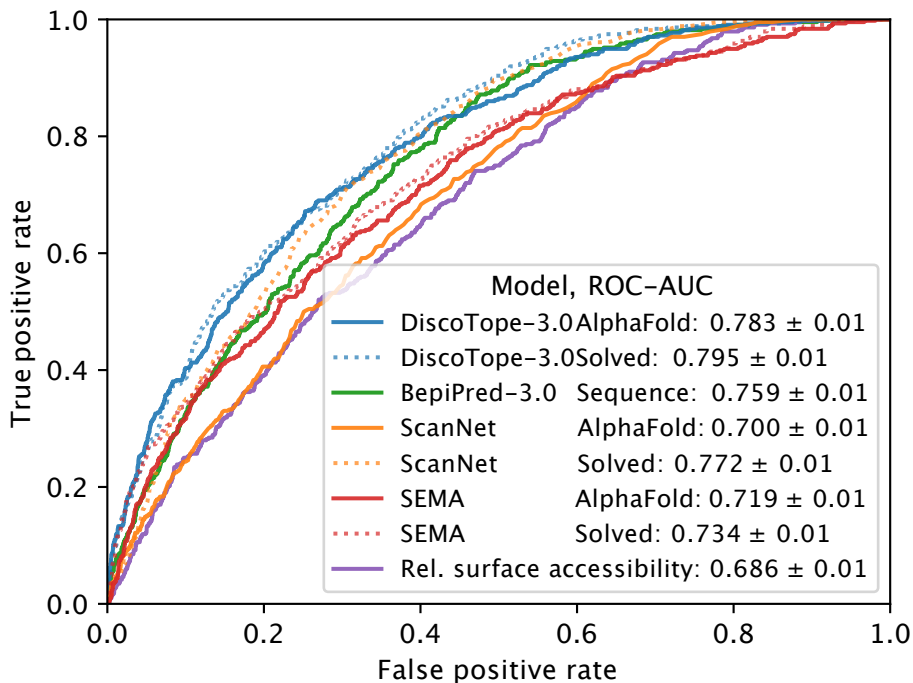

Supplement: Supplementary Figure 1 — Validation set performance up to ensemble size 20. Validation set gain in AUC-ROC from ensembling the full-feature model. Performance graphs are shown for training on either experimentally solved, AlphaFold predicted or both structures, and then evaluated on either the solved or predicted structure validation set. [file DataSheet_1.zip › figures/S2_roc-auc__3.pdf]

a)

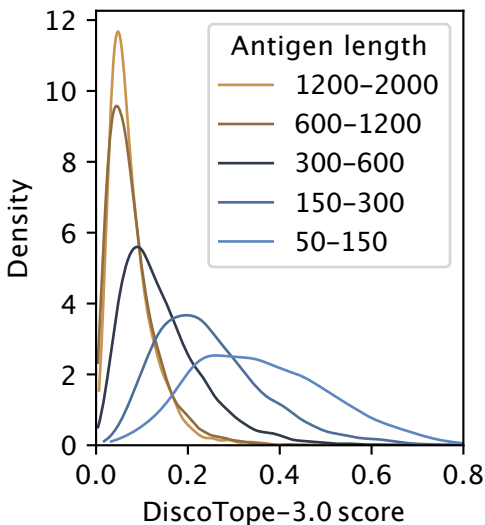

b)

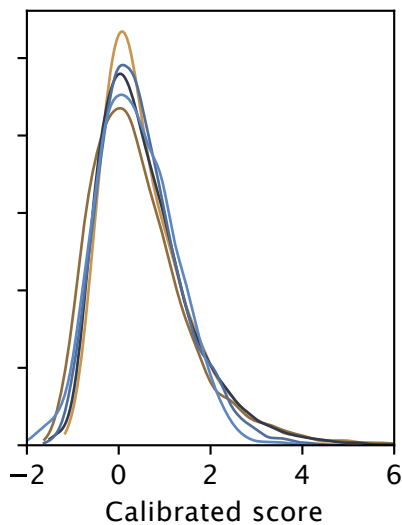

c)

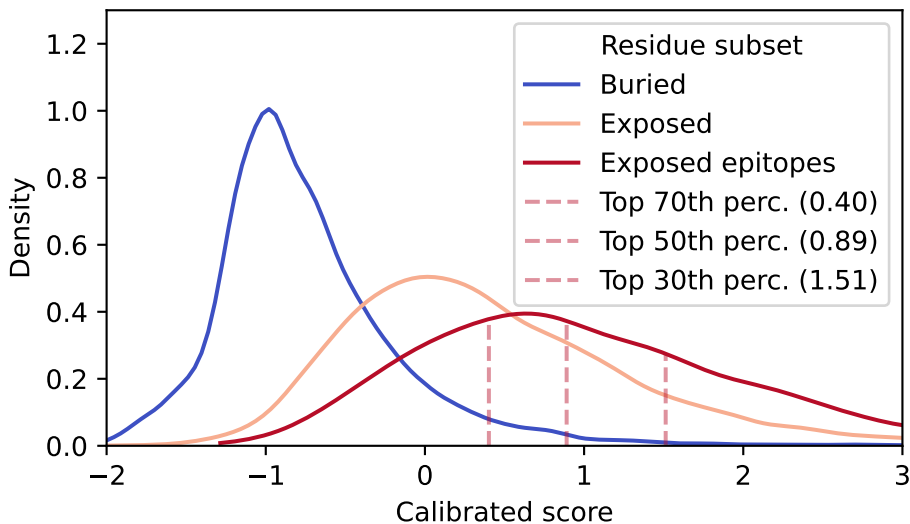

Supplement: Supplementary Figure 1 — Validation set performance up to ensemble size 20. Validation set gain in AUC-ROC from ensembling the full-feature model. Performance graphs are shown for training on either experimentally solved, AlphaFold predicted or both structures, and then evaluated on either the solved or predicted structure validation set. [file DataSheet_1.zip › figures/S7_length_calibration.pdf]

# Lysozyme (n=223 epitopes)

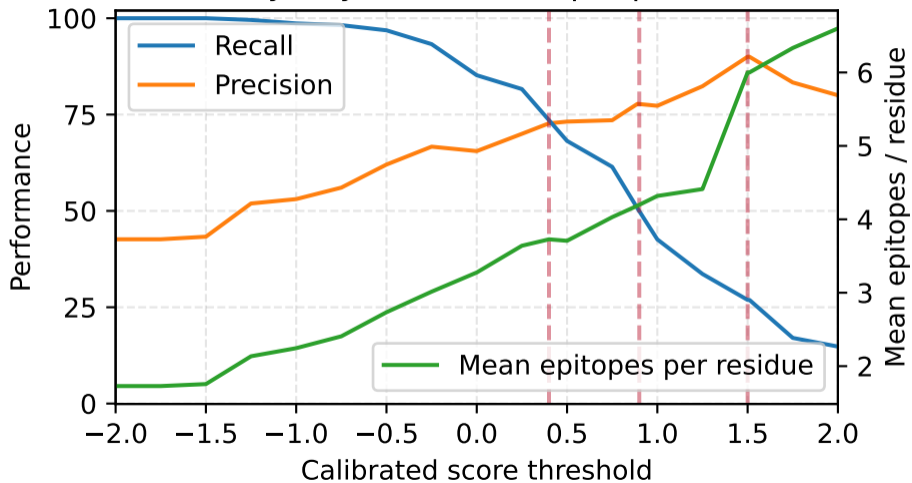

Supplement: Supplementary Figure 1 — Validation set performance up to ensemble size 20. Validation set gain in AUC-ROC from ensembling the full-feature model. Performance graphs are shown for training on either experimentally solved, AlphaFold predicted or both structures, and then evaluated on either the solved or predicted structure validation set. [file DataSheet_1.zip › figures/S8_lysozyme_thresholds.pdf]

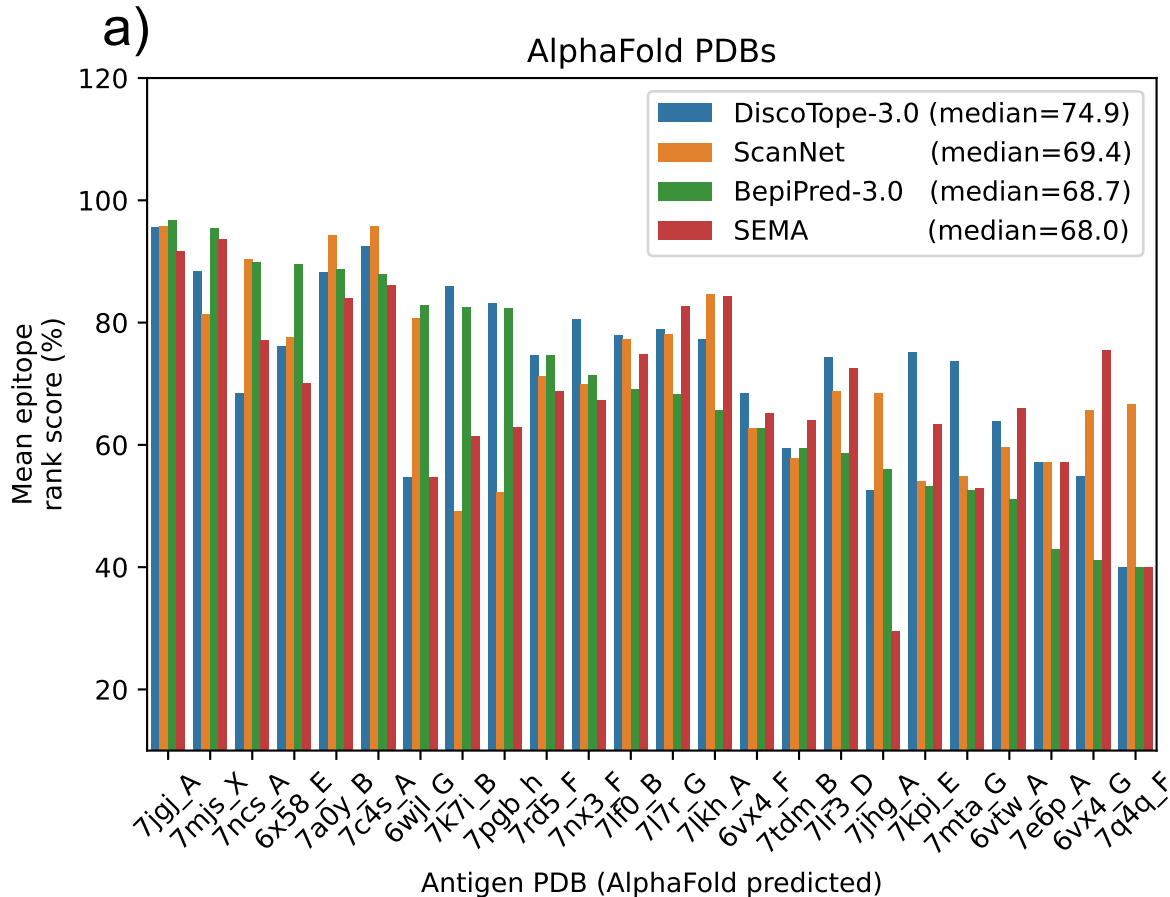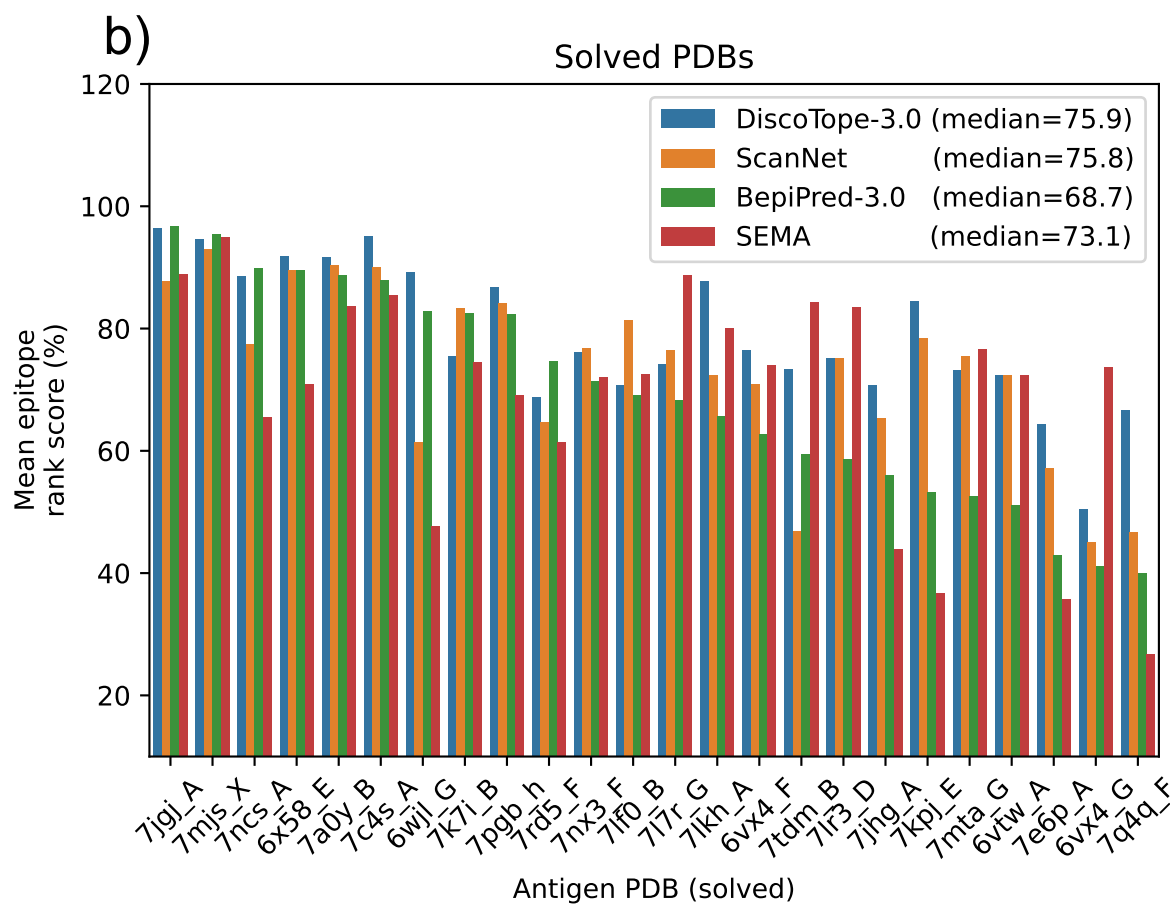

Supplement: Supplementary Figure 1 — Validation set performance up to ensemble size 20. Validation set gain in AUC-ROC from ensembling the full-feature model. Performance graphs are shown for training on either experimentally solved, AlphaFold predicted or both structures, and then evaluated on either the solved or predicted structure validation set. [file DataSheet_1.zip › figures/S3_testset_bargraphs__4.pdf]

### Length to $\mu$ model

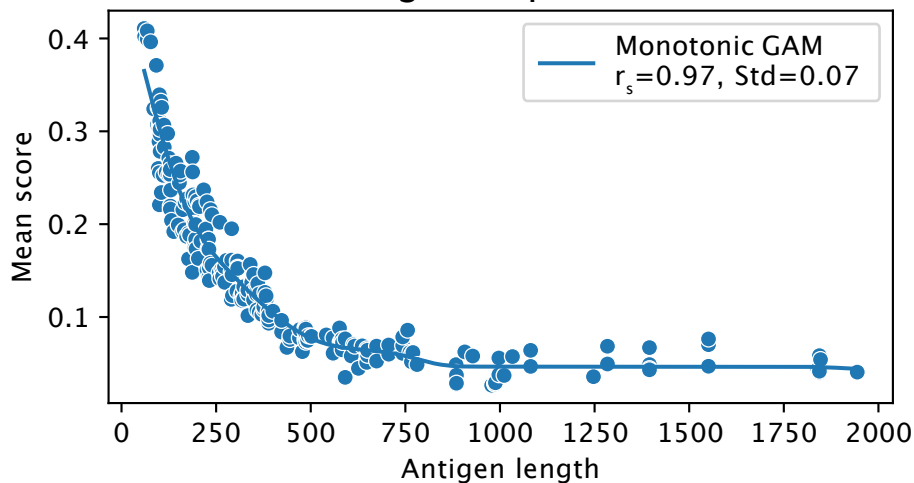

### Surface mean to std model

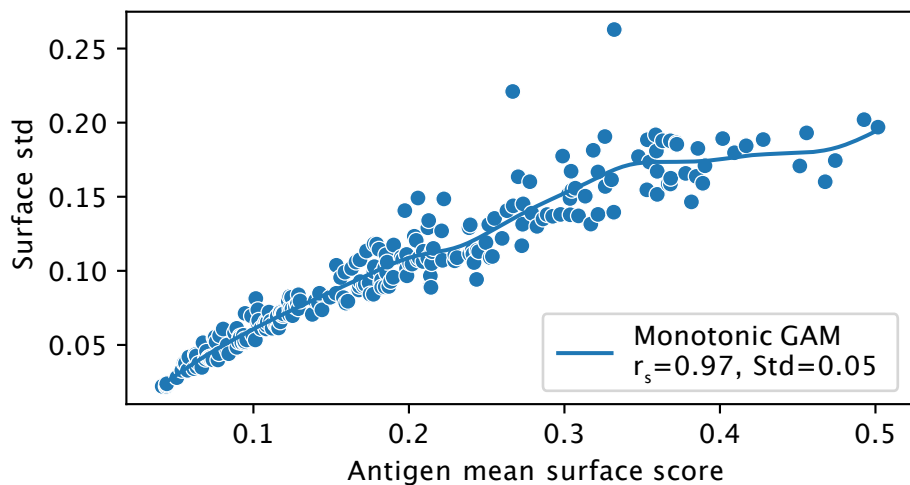

Supplement: Supplementary Figure 1 — Validation set performance up to ensemble size 20. Validation set gain in AUC-ROC from ensembling the full-feature model. Performance graphs are shown for training on either experimentally solved, AlphaFold predicted or both structures, and then evaluated on either the solved or predicted structure validation set. [file DataSheet_1.zip › figures/S9_GAM_models.pdf]

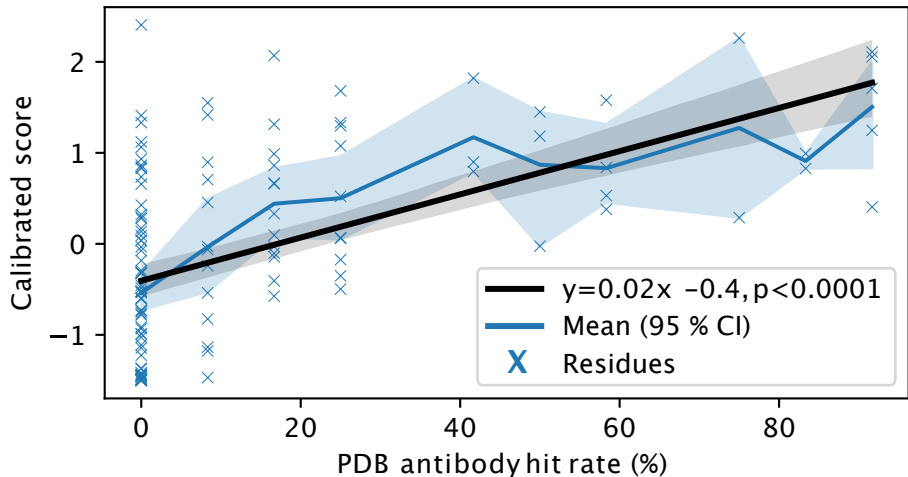

Supplement: Supplementary Figure 1 — Validation set performance up to ensemble size 20. Validation set gain in AUC-ROC from ensembling the full-feature model. Performance graphs are shown for training on either experimentally solved, AlphaFold predicted or both structures, and then evaluated on either the solved or predicted structure validation set. [file DataSheet_1.zip › figures/S6_lyzozyme_pdb_hit_rate__3.pdf]
